# TGDNet: A Multi-Scale Feature Fusion Defect Detection Method for Transparent Industrial Headlight Glass

**DOI:** 10.3390/s25247437

**Published:** 2025-12-06

**Authors:** Zefan Zhang, Jin Tang

**Affiliations:** School of Automation, Central South University, Changsha 410083, China; 224601025@csu.edu.cn

**Keywords:** transparent glass defect detection, dataset enhancing, multi-scale feature fusion, attention mechanism

## Abstract

In industrial production, defect detection for automotive headlight lenses is an essential yet challenging task. Transparent glass defect detection faces several difficulties, including a wide variety of defect shapes and sizes, as well as the challenge of identifying transparent surface defects. To enhance the accuracy and efficiency of this process, we propose a computer vision-based inspection solution utilizing multi-angle lighting. For this task, we collected 2000 automotive headlight images to systematically categorize defects in transparent glass, with the primary defect types being spots, scratches, and abrasions. During data acquisition, we proposed a dataset augmentation method named SWAM to address class imbalance, ultimately generating the Lens Defect Dataset (LDD), which comprises 5532 images across these three main defect categories. Furthermore, we propose a defect detection network named the Transparent Glass Defect Network (TGDNet), designed based on common transparent glass defect types. Within the backbone of TGDNet, we introduced the TGFE module to adaptively extract local features for different defect categories and employed TGD, an improved SK attention mechanism, combined with a spatial attention mechanism to boost the network’s capability in multi-scale feature fusion. Experiments demonstrate that compared to other classical defect detection methods, TGDNet achieves superior performance on the LDD, improving the average detection precision by 6.7% in mAP and 8.9% in mAP50 over the highest-performing baseline algorithm.

## 1. Introduction

As a critical safety component and important exterior element of automobiles, the quality of automotive headlights directly impacts driving safety as well as driving feeling. The transparent glass of headlights must possess excellent optical properties (such as high light transmittance and precise light distribution) to ensure effective illumination. It must exhibit flawless surface quality to meet increasingly stringent aesthetic requirements. Currently, the industry primarily relies on manual visual inspection for quality control. However, manual inspection is highly subjective, uncertain, and inefficient, failing to meet the current industrial demands for high speed and high precision.

Many traditional computer vision methods, such as Gray-Level Covariance Matrix, Gaussian Mixture Markov Random Field, and LBP, have improved detection techniques to some extent. Nevertheless, these methods mainly depend on manual prior knowledge and lack universality and generalization capability, limiting their application in industrial settings. In recent years, deep learning neural network-based algorithms have made significant progress in the field of defect detection, such as for printed circuit boards [1], fabric defects [2], and tire defects [3]. In the domain of glass inspection, methods have also been proposed for materials like 3C glass [4], smartphone glass [5], and aircraft cabin glass [6]. Current methods are mainly divided into supervised methods and unsupervised methods, with supervised methods generally delivering better detection results and thus being more commonly used in industrial inspection.

For training neural networks, the quality of the dataset is crucial, and the image quality within the dataset is particularly important for detection outcomes. Datasets intended for real industrial scenarios need to be collected from actual production environments, where the data acquisition process can interfere with production schedules, making them challenging to obtain. Currently, there are a few datasets for glass defect detection, such as AuGD [7], RSGD, and CGD [8]. Unfortunately, these datasets are often small in scale, and most are based on smartphone screen glass, which is not transparent. Additionally, these datasets suffer from class imbalance issues, making it difficult to train models with satisfactory performance.

To achieve defect detection on transparent glass surfaces, we first collected a total of 2000 images—including both defective and defect-free samples—from factory production lines. Since no relevant dataset existed previously, we employed a novel data augmentation method called the subimage window adaptive matching method (SWAM) to expand our dataset for more effective experimentation. After constructing the dataset, we framed the defect detection task as an object detection problem and proposed a new feature fusion algorithm named TGDNet. This algorithm introduces a novel feature extraction module called TGFE in the backbone, along with an improved attention mechanism named TGD based on the SK attention mechanism. Furthermore, we integrated BiPANet into the neck layer to enhance the effectiveness of feature fusion. We compared TGDNet with several Transformer-based algorithms and achieved promising results in our comparative experiments.

Our contributions can be summarized as follows:

1. A dataset was constructed, and SWAM was developed to enhance class-imbalanced defect images. This process resulted in the LDD, comprising 5532 images with balanced category distribution and real-world applicability.
2. A transparent glass defect detection algorithm named TGDNet was proposed, which includes the TGFE module in the backbone and the TGD attention mechanism. These components enable adaptive feature extraction for irregular small defect targets, improving both detection accuracy and efficiency. They address the challenges posed by diverse defect shapes and inconsistent defect sizes in transparent glass defect detection.
3. Subsequent experiments demonstrate that TGDNet exhibits significant advantages in both detection accuracy and speed compared to multiple classical defect detection algorithms on the LDD.

## 2. Related Works

### 2.1. Dataset Enhancing

Due to the large amount of time and labor costs associated with data collection in industrial settings, datasets manually collected during production are often limited in size and insufficient for large-scale training. Furthermore, in actual production, the quantity of various glass defect categories is influenced by factors such as the production environment, equipment, and materials, leading to significant numerical disparities between categories and resulting in a class imbalance problem. Therefore, using appropriate methods to augment the dataset is of great significance for experiments.

A common method involves copying defective targets and pasting them onto background images. By controlling the number of copy and paste actions for different types of defects, more labeled targets are generated, balancing the quantity of labeled objects across different categories. Kisantal et al. [9] proposed a dataset augmentation framework based on in-image object replication via mirroring. For defective images, the extracted defect subimages are inserted into arbitrary locations within the image. However, due to the limited defect replication operating within a single image, the complexity of the dataset is not significantly improved. Dvornik et al. [10] proposed a dataset augmentation framework that synthesizes object instances and background cross-images. To ensure synthesis quality, a novel neural network was designed to generate context for filtering instances, reducing semantic conflicts between instances and the background. Notably, due to the use of neural networks to generate context, this algorithm has high time complexity.

Unlike copy-based methods, generation-based methods form new samples by mimicking the information structure of existing samples. Shin et al. [11] employed conditional GANs for image-to-image translation to expand medical image datasets while protecting patient privacy. Niu et al. [12] proposed the Surface Defect Generative Adversarial Network (SDGAN), which utilizes two generators. One of the generators produces defective samples from normal samples, while the other restores defective samples to normal ones. This method requires plenty of data in advance. With insufficient datasets and time, GAN is unable to produce high-quality defect images.

### 2.2. Defect Detection

Early studies, based on machine vision and neural network, primarily focused on defect detection on translucent glass. Hou et al. [13] utilized a Gray-Level Co-occurrence Matrix (GLCM) based on LBP to analyze the spectral signature of fluorescent screens for glass defect detection. Luo et al. [14] employed a lightweight model to identify scratches on mobile phone. However, these methods show poor capability in detecting transparent glass and locating the positions of defects.

Over the past few decades, deep learning methods have shown a huge improvement in efficiency and accuracy. R-CNN-integrated region proposals were combined with CNNs to enhance detection accuracy [15]. Faster R-CNN was then developed as an end-to-end pipeline and sped up region proposal generation [16]. To reduce the proposal step, single-stage detectors were introduced, including YOLO series and ATSS [17]. These methods enable real-time solutions.

YOLO series are widely used in industry. Taking YOLOv8 as an example [18], the module composition and functions of a single-stage object detection network are detailed. YOLOv8 is a state-of-the-art, single-stage object detection framework utilized in industry, integrating the advantages of multiple YOLO versions and other deep learning models. Similarly to YOLOv5, YOLOv8 utilizes CSPDarknet and optimizes it into an more advanced module. The backbone of CSPDarknet in YOLOv8 was improved from the C3 to the C2f, making it more lightweight and more flexible (three convolutions to two convolutions) [19]. After feature extraction, YOLOv8 employs the SPPF module to conduct pooling operations over the spatial dimensions of feature pyramids, fusing global information across larger receptive fields. This module performs pooling on features of different scales to effectively aggregate information from various feature layers. Furthermore, YOLOv8 utilizes PANet for bidirectional feature fusion, enhancing its ability to process and integrate information across different feature levels [20].

Due to the powerful capabilities of YOLOv8, it and its related version are widely used in different kinds of detection tasks [21,22,23,24,25]. Furthermore, some Transformer-based models have incorporated attention mechanisms to enhance their capability to detect objects at various scales [26,27]. Meanwhile, some researchers have also started working on lightweight modifications tailored to specific types of defects. Recently, Ji et al. proposed LCCD-Net [28], which employs a lightweight algorithm capable of rapidly detecting defects on cigarettes. However, the field of defect detection in transparent glass still suffers from the limited availability of data and research methods, as well as the lack of large-scale, high-quality defect datasets in this domain.

### 2.3. Attention Mechanism

Attention mechanisms mimic the human visual system’s selective focus on information, enabling models to dynamically concentrate on more critical parts of the input, thereby simulating defect detection. The Squeeze-and-Excitation (SE) attention mechanism is one of the most classic variants [29], which allows the network to adaptively recalibrate channel-wise feature responses by explicitly modeling interdependencies between channels. The Convolutional Block Attention Module (CBAM) sequentially applies channel and spatial attention [30], optimizing features based on both. The Selective Kernel (SK) attention primarily involves three operations: split, fuse, and select. It captures features at different scales through multiple convolutional branches with different kernel sizes, then adaptively integrates these branches via an attention mechanism [31]. The SPPF module in YOLOv8 may experience reduced detection efficiency due to the influence of large convolutional kernels. The principle of the Selective Kernel (SK) attention mechanism can be effectively leveraged within the convolutional kernel functions of YOLOv8 to enhance the detection efficiency of the network framework.

## 3. Methodology

### 3.1. Overview

Currently, there is a scarcity of defect image datasets for transparent glass, with the main defect types being spots, scratches, and abrasions. Therefore, we first collected 2000 images of automotive headlight lenses. Due to the limited number of certain defect types in production and the imbalance in sample quantities across different defect categories, we applied SWAM to replicate specific defects, ultimately obtaining a balanced dataset comprising 5532 images. Furthermore, to enhance the efficiency and speed of manual inspection in industrial production, we proposed a multi-scale feature fusion network for screening defective products.

### 3.2. Dataset Preparation and Lighting Configuration

**Figure 1:**
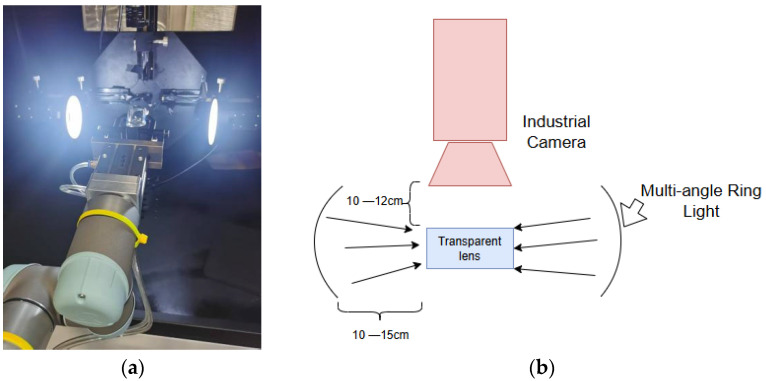
Equipment for lens defect dataset: (**a**) equipment, (**b**) diagram of equipment.

In glass inspection tasks, the lighting configuration critically impacts image quality, making the selection of an appropriate lighting method crucial. For defect detection in transparent glass, surface defects such as scratches and abrasions are often transparent. These kinds of defects typically require manual rotation of the glass, so research focuses on observing them from specific angles through a specific light source. There are several lighting methods in the defect detection field; however, only one method is especially useful for transparent glass. The other approaches often cause reflections or overly bright backgrounds, compromising the detection of surface defects. Bright-field illumination causes specular reflection on transparent glass, making the area to be inspected unobservable. Backlighting will cause the transparent glass to appear completely white, making colorless defects such as scratches and abrasions on the specular surface undetectable. Therefore, we developed an automated inspection system that employs bidirectional multi-angle ring lighting arranged for dark-field forward illumination. This setup effectively reveals surface scratches, abrasions, and spots in transparent glass. The configuration is illustrated in Figure 1.

**Figure 2:**
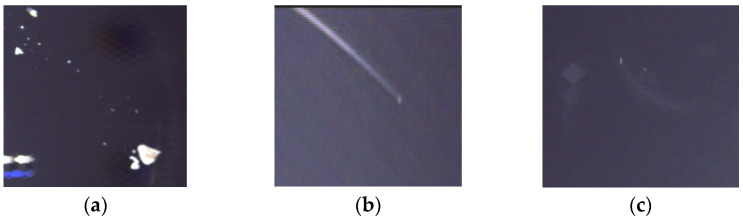
Three different kinds of transparent glass defects: (**a**) spot, (**b**) scratch, (**c**) abrasion.

Figure 1 illustrates a dark-field forward illumination configuration. The distance between the light source and transparent glass is changeable and mostly kept at 10–15 cm in our experiment. For transparent glass, using bright-field forward illumination would generate excessive specular reflection, which severely impairs the inspection of the target area. Conversely, back lighting would result in a uniformly bright field, causing transparent defects to lack contrast and become undetectable. Therefore, the angle from the top of the multi-angle ring light source to the vertex of the lens generally does not exceed 20 degrees. Under the dark-field forward illumination shown, which employs side lighting, most specular reflections are directed away from the camera. This optical arrangement causes light to scatter at surface defects, making them clearly visible to the camera. The three main types of these defects are illustrated in Figure 2.

**Figure 3:**
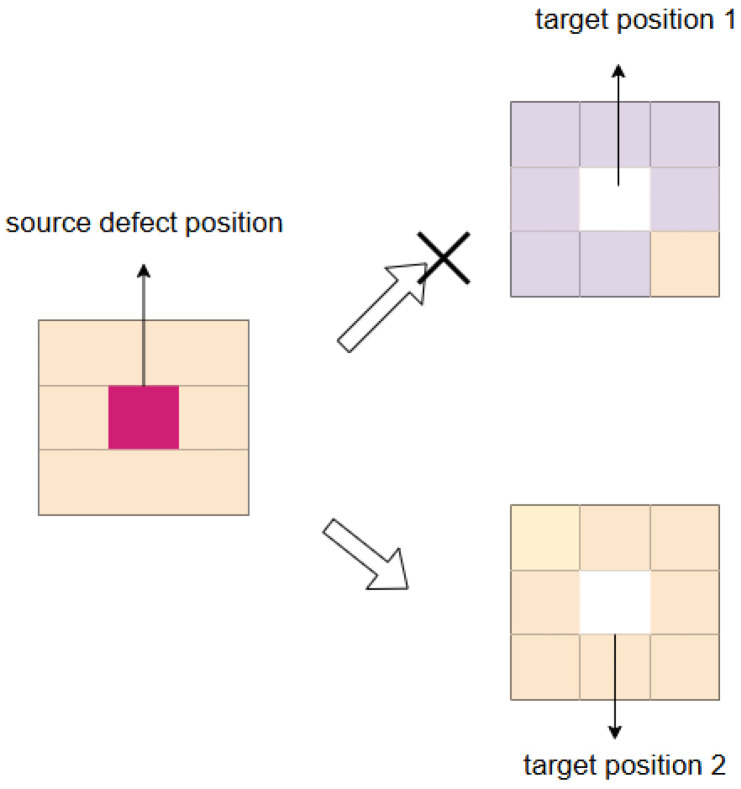
Diagram of defect copying process. The difference between the neighborhood of the target position determines whether it can be copied to this position.

Figure 2 displays the three defect categories in the dataset: spots, scratches, and abrasions. The initial dataset was constructed, comprising 950 defect-free background images and 1050 defect-containing images. Among these, images with spot defects are more numerous, while the number of abrasion defects is significantly lower than the other two types. To balance the quantity of each defect category, enrich the dataset’s diversity, and ensure data validity, we adopted a window cropping method to replicate target defects. Firstly, SWAM divided the images into several parts according to a specific window size; then the window extracted subimages containing the target defects, which then became the target subimages. After that, we copied these subimages onto the selected background and converted the background images into new images containing the target defects. Specifically, both the defect subimages and background images were sourced from the training set of the dataset. Based on the proportion of different defect types, more labeled defects were generated. To ensure that the defect subimages do not cause semantic conflicts with the background and can be integrated well, similarity comparisons between the target position and its neighborhood are necessary. These similarity comparisons include luminance similarity and contrast similarity. The environment similarity can be defined as follows:

(1) $$S_{env}=\frac{1}{8}\sum_{i=1}^{8}{SIM(Sn}_{i},Tn_{i})$$

where $Sn_{i}$ is the *i*th neighborhood around the source defect position, $Tn_{i}$ is the *i*th neighborhood around the target position, and  SIM is the luminance similarity and contrast similarity, which is defined as

(2) $$\;\;\;SIM(Sn,Tn)=\frac{\left(2\mu_{Sn}\mu_{Tn}+d_{1}^{2})(2\sigma_{SnTn}+d_{2}^{2}\right)}{\left(\mu_{Sn}^{2}+\mu_{Tn}^{2}+d_{1}^{2})(\sigma_{Sn}^{2}+\sigma_{Tn}^{2}+d_{2}^{2}\right)}$$

where $\mu_{Sn}$ and $\mu_{Tn}$ are the averages of Sn and Tn, respectively; $\sigma_{Sn}^{2}$ and $\sigma_{Tn}^{2}$ are the variances of Sn and Tn, respectively; $\sigma_{SnTn}$ is the covariance between Sn and Tn; and $d_{1}$ and $d_{2}$ are generally set to 0.01 and 0.03, respectively. When $S_{env},$ ranging from 0 to 1, is smaller than 0.7, the difference between the neighborhood of the target position and the source defect position is too large, so it is unsuitable for copying the defect. When $S_{env}$ is above 0.7, the defect is suitable to be copied to the target position. The defect will be pasted into the target position. The process can be described as shown in Figure 3.

**Figure 4:**
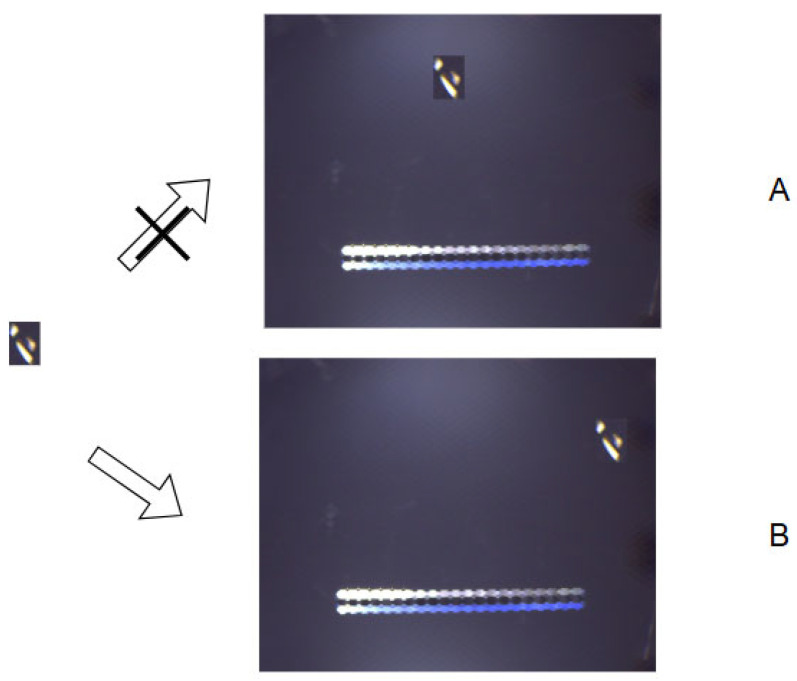
Result of defect copy process. (**A**) shows an inappropriate position for subimage and (**B**) shows an appropriate one. The subimage can only be copied to specific target positions based on its neighborhood.

In this process, target position 1 has low environment similarity, the defect is not suitable for this position. On the other hand, the neighborhood of target position 2 has more similarity with the neighborhood of the source defect position. Through this method, we manage to obtain a series of capable target positions and achieve data augmentation for insufficient defective samples. Figure 4 demonstrates the visual effects of subimage replacement in different backgrounds. In the process, the defect cannot be copied to background A, where the surrounding environment is too distant from the defect itself, while background B proves to be a suitable alternative. In this way, defects with limited quantity and variety can be replicated to appropriate positions, effectively enhancing the data.

### 3.3. TGDNet

**Figure 5:**
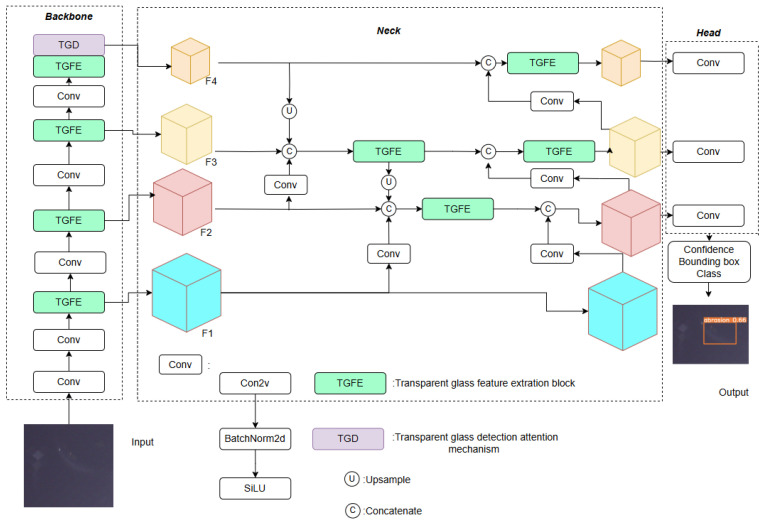
Diagram of TGDNet.

TGDNet is designed to automatically identify and localize glass defect areas in captured images. Our approach is built upon a single-stage detection framework and consists of three core components: the backbone, neck, and head. The backbone handles feature extraction, the neck performs feature fusion, and the head predicts the locations and categories of defects. The detailed architecture of TGDNet is illustrated in Figure 5.

As illustrated in Figure 5, the input image is first processed by the backbone. Through the lightweight linear deformable convolution in the TGFE module, the computational flow is streamlined, and four feature layers (F1, F2, F3, F4) are extracted. By incorporating the TGD attention mechanism, the model effectively extracts and selects information from receptive fields of varying sizes, thereby enhancing the detection performance for defects of different scales. In the neck, we employ BiPANet, an improved PANet which integrates shallow and deep features to capture high-resolution detection information. Compared to the original module, BiPANet improves detection accuracy while reducing computational time. Finally, the detection results are generated by the head.

#### 3.3.1. TGFE Block

**Figure 6:**
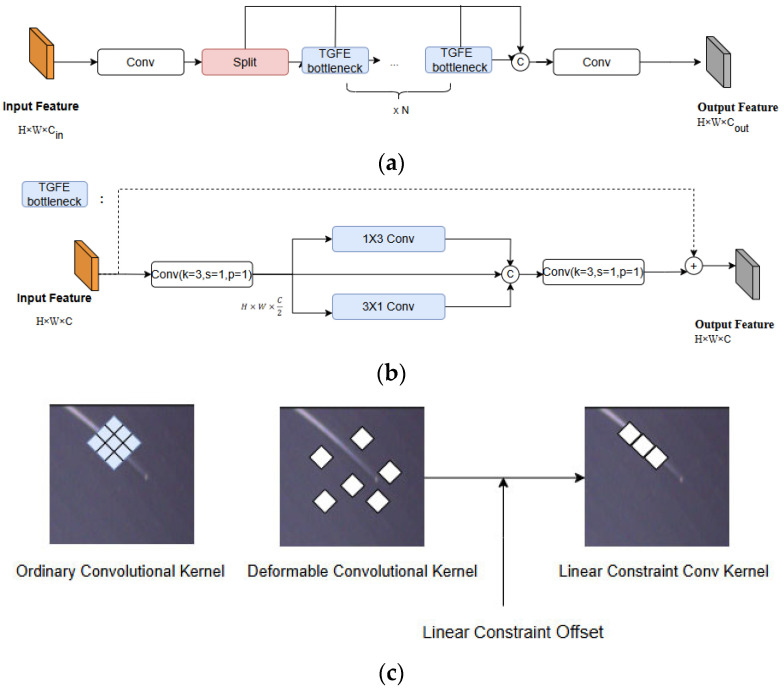
Diagram of (**a**) TGFE block and (**b**) TGFE bottleneck. (**c**) Difference between ordinary convolutional kernel and Linear Constraint Convolutional Kernel.

As a classical approach, CNN typically employs fixed-size convolutional kernels to handle defects of varying sizes. However, given that a significant proportion of defects on transparent glass are small or elongated (spots and scratches), traditional convolution methods often lead to unnecessary computational overhead. To address this, we propose a feature extraction structure called the TGFE block. The diagram of TGFE is shown in Figure 6a,b.

The design of TGFE seems much more realistic compared to C2f. However, the difference between the two bottlenecks great influences this network. TGFE introduces deformable convolution. Compared to standard convolution, deformable convolution adapts to target shapes by learning offsets, enhancing the model’s ability to extract fine-grained local features across variously shaped defects. Deformable convolution commonly constrains offsets only in the relative receptive field range, which leads to high computational costs and unnecessary adjustments that may overlook slender defect structures. To better align deformable convolution with the free-form linear structures of transparent glass defects, we introduce linear offset constraints to regulate the deformable convolution.

In Figure 6c, the TGFE block utilizes two kinds of linear deformable convolutional kernels (1 × 3 and 3 × 1) to extract features along two vertical directions, respectively. By fixing the center of the convolutional kernel and comparing the offsets of other grid points relative to this center, the TGFE block can determine whether to apply linear continuity constraints to reduce the computational load. Designed specifically for transparent glass defect detection, this module maintains the capability to identify defects with complex shapes while incorporating a lightweight linear constraint convolution method adapted for elongated defects. This approach reduces computational overhead and detection time, thereby improving the overall detection efficiency for this specific defect category.

#### 3.3.2. Transparent Glass Defect Attention Mechanism

Transparent glass defects are typically small objects. However, the traditional SPPF module employs large convolutional kernels for multi-scale feature extraction, which is computationally inefficient. The principle of the Selective Kernel (SK) attention mechanism inspires a more flexible approach to utilizing convolutional layers, thereby enhancing the framework’s detection efficiency. The core strength of the SK mechanism lies in its ability to maintain multiple convolutional branches with different kernel sizes in parallel and then adaptively fuse their outputs. This allows the network to dynamically select the most appropriate receptive field for different inputs. By incorporating this adaptive multi-kernel fusion strategy into the backbone, the network can intelligently prioritize smaller, faster kernels where sufficient, reserving the more computationally intensive larger kernels only when necessary to capture broader context. This results in a more efficient allocation of computational resources, improving overall detection speed without compromising the richness of multi-scale feature representation.

**Figure 7:**
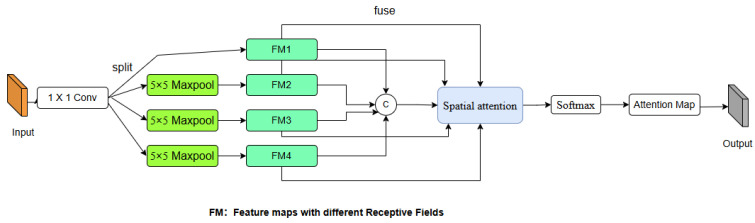
Diagram of TGD attention mechanism.

Building upon the SK concept, we propose a novel attention mechanism termed TGD, tailored for our specific task. The architecture of the TGD mechanism is illustrated in Figure 7 as follows:

Specifically, our approach adopts the SPPF architecture through a series of convolutional and max pooling layers to capture multi-scale features with varying receptive fields (1 × 1, 5 × 5, 9 × 9, and 13 × 13) from the input feature map. First, a 1 × 1 convolutional layer reduces the channel depth to half of the original input for computational efficiency. This is followed by three successive 5 × 5 max pooling layers that progressively extract features across increasing receptive fields. The resulting multi-scale feature maps, denoted as $FM_{i}$(i = 1, 2, 3, 4), are then fused via a spatial attention mechanism. This mechanism assigns adaptive weights to features at different scales, allowing the model to dynamically prioritize the most informative representations. The multi-scale features are first concatenated into a composite feature $F_{s},$ which is then processed through the spatial attention module and a Softmax function to generate channel-wise spatial attention weights, $W_{s}$. The spatial attention weights of each channel correspond to the four input features. Finally, the attention map $A_{m}$ is obtained by performing element-wise multiplication between the feature maps and their corresponding attention weights, followed by a summation operation. $A_{m}$ effectively aggregates the most salient information across all scales. The overall process can be formulated as follows:

(3) $${\;\;\;\;\;\;\;\;\;\;\;\;F}_{s}=Concat\left({FM}_{i}\right),\;i\;=\;1,2,3,4$$

(4) $${\;\;\;\;\;\;\;\;\;\;\;\;\;\;\;\;\;\;\;\;\;\;\;\;\;\;\;W}_{s}=Softmax(Conv_{7\times 7}(Concat(AP_{global},MP_{global}))$$

(5) $$A_{m}=Sum\left({FM}_{i}⊗W_{s}\right),\;i=1,2,3,4$$

where $AP_{global}$ and $MP_{global}$ represent the global average pooling operation and the global max pooling operation across the channel dimension, respectively. ⊗ denotes element-wise multiplication. The spatial attention weights multiply with the features in corresponding channels. The result is multiplied element-wise with the attention map to obtain the output of the TGD attention mechanism. Overall, the TGD attention mechanism equips the network with an enhanced capability to focus on specific small-sized objects, which is of great importance in transparent glass detection.

#### 3.3.3. Neck of Network

To enhance the capability of small object detection, we optimized the PANet in the neck to BiPANet, thereby strengthening feature fusion across different layers. In a typical object detection model like YOLO, BiPANet is integrated into the network as the “Neck”, situated between the “Backbone” and the “Head”. Replacing a simpler neck (e.g., a standard FPN or PANet) with BiPANet gives the model a significant boost in detection accuracy, especially on complex datasets with multi-scale objects, without a prohibitive increase in computational cost.

**Figure 8:**
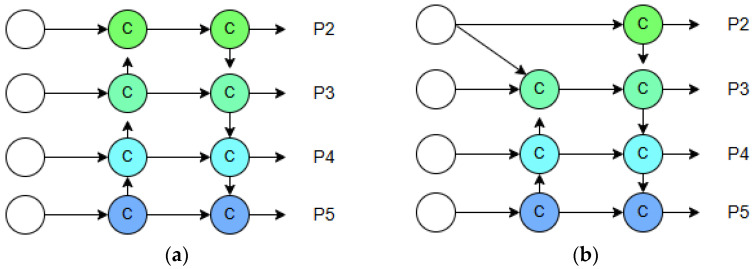
Diagram of (**a**) PANet and (**b**) BiPANet.

As shown in Figure 8, compared to PANet, BiPANet not only merges features from adjacent and higher layers but also incorporates output features from lower layers. This design integrates low-level features with minimal computational overhead while preserving their corresponding spatial details.

In summary, BiPANet is a powerful feature fusion architecture that leverages iterative, bidirectional information flow to build a comprehensive and highly effective multi-scale feature representation. This makes it particularly suited for challenging detection tasks in industrial and high-precision applications.

## 4. Experiments

### 4.1. Evaluation Metrics

For empirical evaluation, we employ precision (P, recall (R), F1-score (F1), and mean average precision (mAP50, mAP50:95)) to quantify detection accuracy. Meanwhile, frames per second (FPS), the number of parameters (Params), and runtime are used to assess detection efficiency. All predictions are categorized into True Positives (TPs), False Positives (FPs), True Negatives (TNs), and False Negatives (FNs). Precision, recall, F1-score, mAP, and FPS are defined as follows:

(6) $$\;\;P\;=\;\frac{TP}{TP+FP}\;\;\;\;\;\;\;\;\;\;$$

(7) $$\;R=\frac{TP}{TP+FN}$$

(8) $$F1=2\frac{P\times R}{P+R}$$

(9) $$AP=\sum_{i=1}^{n-1}\left(r_{i+1}-r_{i}\right)P_{inter}(r_{i}+1)$$

(10) $$\;\;\;mAP=\frac{1}{C}\sum_{i=1}^{C}AP_{i}$$

(11) $$f=\frac{1}{t}$$

where $P_{inter}$ denotes the interpolated P. $r_{i}$ denotes the R value corresponding to the first interpolation of the P interpolation segment in ascending order. C denotes the number of categories. t means the average time required to process one image.

The mean average precision (mAP) serves as a core evaluation metric by synthesizing both precision and recall, thereby quantifying the trade-off between identification correctness and completeness. Since mAP values are sensitive to the IoU threshold, we report results under different criteria: AP50, AP75, and AP50:95 (which averages mAP over IoU thresholds from 0.5 to 0.95 in steps of 0.05). The last one, AP50:95, is commonly referred to as the standard mAP. This multi-threshold evaluation provides a comprehensive overview of model performance across varying localization strictness. In this work, mAP, evaluated specifically on small objects (mAP_s), is adopted as the key accuracy criterion. A small object is defined as having a resolution below 32 × 32 pixels.

Considering the industrial requirement for real-time processing, we compare our approach with representative single-stage (YOLO series) and two-stage (Faster R-CNN) detectors. For a fair comparison, all models are trained with the AdamW optimizer (learning rate = 0.001, beta_1_ = 0.9, weight decay = 0.01). YOLO-based models are trained with a batch size of 32 for 150 epochs at an input resolution of 640 × 640. All experiments use medium-sized architectures with unified channel settings to ensure a balanced comparison between efficiency and accuracy.

### 4.2. Implementation Details

The experiments were conducted on a setup with 4 RTX 3080 GPUs using PyTorch 1.10. During the construction of the LDD, we employed a 150 × 150-pixel sliding window with a stride of half the window size to generate subimages. The dataset was then split into training, validation, and test sets in a ratio of 7:2:1. Within each set, the defect instances were distributed as follows: spots accounted for 40%, while scratches and abrasions each constituted 30%.

### 4.3. Experiment Results

#### 4.3.1. Accuracy Comparison

In the experiment, we employ Faster RCNN and YOLO series models, from the classic one to the newest one. All these model were trained with the same metrics to create a truly accurate result. The result is shown in Table 1.

This result shows that TGDNet has a great advantage over the other models in terms of P and mAPs. The precision of TGDNet is 6.5% over YOLOv5 and its mAP is 5.5% over YOLOv8. Meanwhile, it is a little ahead of other model in F1 as well as mAP_50_. In summary, TGDNet demonstrates exceptionally outstanding detection accuracy in transparent glass detection. Meanwhile, it can be observed that the newer detection models, YOLOv10 and YOLOv11, exhibit even lower detection accuracy compared to YOLOv8.

#### 4.3.2. Efficiency Comparison

The efficiency of the model can be described in three dimensions: f, Params, and FLOPs. The result is shown in Table 2.

Due to the need for dynamic convolution computations, the TGDNet model must process more parameters and incur longer computation times. However, compared to Faster RCNN, TGDNet has significantly fewer parameters and achieves faster image detection processing speed. Relative to other YOLO algorithms, TGDNet only increases the number of parameters by 12.4 M compared to the most lightweight YOLOv11 model while maintaining a processing speed of 71.1 frames per second. This faster processing time enables it to meet the requirements for real-time detection functionality.

#### 4.3.3. Ablation Experiment

**Figure 9:**
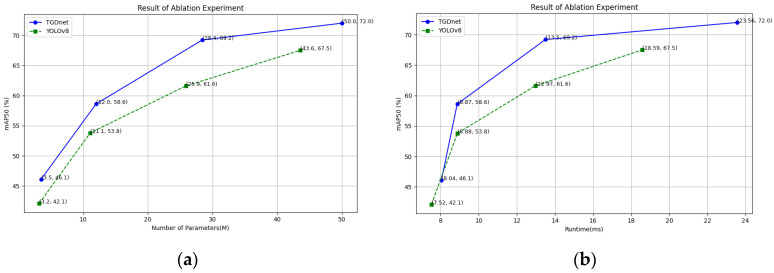
Ablation experiment on Params and runtimes. The relationship between (**a**) Params and mAP_50_; (**b**)runtime and mAP_50_.

Based on our previous experimental findings, YOLOv8 and YOLOv5 demonstrate relatively superior detection accuracy and efficiency on the LDD compared to the other models. To visually demonstrate the effectiveness of the improved module in TGDNet, we conducted a comparative experiment between TGDNet and YOLOv8, examining the relationship between the number of model parameters, detection runtime, and detection accuracy. The result is shown in Figure 9.

We conducted experimental comparisons on four model sizes (n, s, m, l) to evaluate the actual performance of the two models. The results demonstrate that our method outperforms YOLOv8 across all model sizes. Furthermore, the findings indicate that increasing the model size significantly improves detection accuracy. Depending on the specific requirements for detection accuracy and efficiency, different model sizes can be selected accordingly.

To demonstrate the role of each module in our network, we conducted another experiment involving an ablation study of the network components. The purpose of this study is to observe the specific impact of each module on the detection accuracy and results. We evaluated the functionality of each module from multiple dimensions, including precision (P), recall (R), F1-score (F1), mAP50, mAP, number of parameters (Params), FLOPs, and f. The result is shown in Table 3.

From the results, it can be observed that the absence of the TGFE module significantly impacts the model’s detection accuracy. Although this module increases number of parameters to some extent, the 3.4% decrease in precision (P) and 3.7% reduction in mAP50 outweigh the benefits, making the trade-off unfavorable. This experiment demonstrates the critical importance of the TGDN module in the detection network for this dataset. The TGD attention mechanism also has a notable influence on detection accuracy. When replaced with the traditional SPPF module, the network’s sensitivity to transparent glass detection noticeably decreases. Regarding the BiPANet module, replacing BiPANet with the conventional PANet also leads to a decline in detection accuracy, with precision (P) decreasing by 2.7% and mAP50 dropping by 1.9%. This confirms that BiPANet can better integrate and preserve features from different layers, thereby aiding in the detection of small targets. In summary, the ablation study confirms that each module plays a critical role in the defect detection results.

#### 4.3.4. Visualization

**Figure 10:**
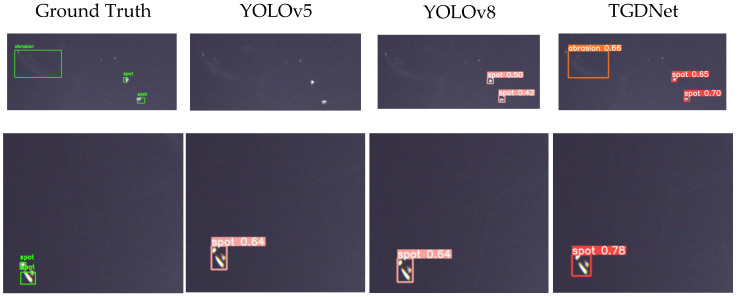
The visualization of defect detection on LDD. The four columns of images from left to right correspond to the detection results of the ground truth, YOLOv5, YOLOv8, and TGDNet, respectively.

Figure 10 shows us the ground truth and some of the visualization results from the top three most accurate methods. The visualization results show us that TGDNet shows a great advantage for small object detection and blurred defects in images. Usually, abrasion is caused by some tiny operational error, so it is even harder to detect compared to a scratch. As we all know, some kinds of scratches are difficult to notice; even a skilled worker might sometimes ignore them.

TGDNet demonstrates high precision in capturing minute and subtle defects while also effectively identifying those that blend into the background. Moreover, it yields higher confidence scores for targets that are consistently detected by the majority of the network. Consequently, the algorithm is particularly suitable for detecting small defects and holds significant promise for future industrial applications in transparent glass inspection.

Overall, experiments on accuracy and efficiency, alongside visualization results, validate that TGDNet achieves superior detection performance while maintaining a lightweight architecture. Its outstanding detection capabilities enable various application scenarios: quality inspection of glass products during manufacturing, safety monitoring of glass in use, and value assessment of glass for recycling. Furthermore, in the era of general-purpose object detection networks, TGDNet and its shape and size perception modules effectively address the unique challenges of glass defect detection. This approach enhances the detection model with domain-specific features, boosts its performance on glass defects, and holds potential for extension to other specialized detection tasks.

## 5. Conclusions

In summary, we utilized SWAM to create and refine a category-balanced transparent glass defect dataset and, based on this, developed an algorithm named TGDNet specifically designed for small object detection. Comparative experiments and ablation studies demonstrate that its modules, TGFE and TGD, significantly enhance detection accuracy for small defects in transparent glass. The visualization results further confirm that the algorithm improves detection precision for small targets and, under comparable detection capability, the enhanced algorithm yields detection outcomes with higher confidence scores.

## Figures and Tables

**Table 1:** Quantitative experiment results of detection accuracy comparison across models.

Model	P (%)	R (%)	F1 (%)	mAP_50_ (%)	mAPs (%)
Faster RCNN	69.1	73.2	71.1	60.9	40.9
YOLOv5	74.3	69.0	72.5	60.0	42.2
YOLOX	69.0	72.1	70.4	56.6	34.8
YOLOv8	74.0	**77.9**	75.0	61.6	43.1
YOLOv10	70.6	72.4	71.2	59.8	41.9
YOLOv11	66.7	73.0	70.4	58.5	39.3
TGDNet	**80.8**	75.5	**77.8**	**68.7**	**48.6**

Note: the bold denotes the best performance, while the underlined text represents the second-best performance.

**Table 2:** Quantitative experiment results of detection efficiency comparison across models.

Model	f (img/s)	Params (M)	FLOPs (G)
Faster RCNN	41.2	40.1	197.1
YOLOv5	73.2	25.5	70.2
YOLOX	70.0	22.3	75.2
YOLOv8	79.5	25.9	80.0
YOLOv11	**82.1**	**16.0**	**60.1**
TGDNet	71.1	28.4	65.8

Note: the bold denotes the best performance, while the underlined text represents the second-best performance.

**Table 3:** Ablation experiment results of TGDNet.

Model	TGFE	TGD	BiPANet	P (%)	R (%)	F1 (%)	mAP50 (%)	mAPs (%)	Params (M)	FLOPs (G)	f (img/s)
Baseline	×	×	×	73.9	56.1	63.5	61.6	41.7	**21.8**	75.2	**78.1**
M1	√	×	×	77.3	58.3	65.2	65.3	47.2	26.0	95.2	62.9
M2	√	√	×	78.1	**76.5**	76.0	66.8	48.1	26.6	95.9	58.5
TGDNet	√	√	√	**80.8**	75.5	**77.8**	**68.7**	**48.6**	26.1	**65.8**	71.1

## Data Availability

The data supporting the findings of this study can be applied from authors.
